# Sex Differences in Bone, Muscle, and Inflammatory Markers and Their Associations with Muscle Performance Variables

**DOI:** 10.3390/sports11110215

**Published:** 2023-11-06

**Authors:** Pragya Sharma Ghimire, Adam Eckart, Ibtihal K. Al-Makhzoomy, James Stavitz

**Affiliations:** College of Health Professions and Human Services, Kean University, 1000 Morris Ave, Union, NJ 07083, USA; eckarta@kean.edu (A.E.); ialmakhz@kean.edu (I.K.A.-M.); jstavitz@kean.edu (J.S.)

**Keywords:** Wnt signaling, inflammation, muscle performance, sex difference

## Abstract

The importance of various markers such as Sclerostin, Dickkopf-1 (DKK-1), Irisin, receptor activator of NF-kB ligand (RANKL), and Vitamin D have been well studied in bone metabolism. Additionally, inflammatory cytokines such as tumor necrosis factor-alpha (TNF-α) and Interleukin 6 (IL-6) have been shown to hinder muscle protein synthesis, leading to the loss of muscle and strength. However, a research gap exists in understanding their role in muscle function and physical activity. Therefore, this study aims to explore the serum levels of Sclerostin, DKK-1, Irisin, IL-6, RANKL, Vitamin D, and TNF-α and assess their relationships with upper- and lower-body strength in young adults. In this study, 38 college-aged students (18–23 years), males and females, participated and completed the protocols. The participants’ lower and upper body strength were assessed by the vertical jump test (Just Jump, Probotic, AL) with a Tendo FitroDyne (Tendo Sports Machines, Trencin, Slovak Republic) and handgrip (HG) dynamometry (Takei Scientific Instruments, Yashiroda, Japan), respectively. Fasting morning blood samples were analyzed for serum levels of biomarkers by ELISA. The results indicate significant sex differences in Sclerostin, DKK-1, Irisin, and Vitamin D levels (*p* < 0.05). Furthermore, a positive association was observed between Sclerostin, DKK-1, and Vitamin D, with lower body muscle performance variables (*p* < 0.05). Conversely, a significant negative correlation was observed between TNF-α and lower-body muscle performance variables (*p* < 0.05). The results suggest that these markers may have a distinct effect on muscle performance, underscoring the need for further investigation to elucidate the concept of muscle–bone crosstalk.

## 1. Introduction

Muscle and bone tissues share a common somatic mesodermal origin and constitute musculoskeletal functional units for movement. In addition, they may function in Calcium reservoirs, protect the internal organs, and maintain glucose homeostasis, possibly with a common molecular network [1,2]. Mechanostat theory suggests mechanical loading impacts bone mass and geometry, while evidence suggests that muscle force can drive bone adaptivity responses [3]. Previous evidence suggests a positive correlation between muscle mass and bone mineral density (BMD), indicating that increased muscle mass is linked to higher BMD. Conversely, the loss of muscle mass associated with aging could contribute to bone loss [2,3]. Furthermore, pathological states such as Vitamin D deficiency and glucocorticoid imbalance can exacerbate bone and muscle loss. Consequently, it is essential to understand the reciprocal phenomenon between bone mass, geometry, and muscle mass to characterize functional units for movement and enhance physical health in humans [4,5].

Many overlapping signaling pathways, including Wnt, Hedgehog, Growth Hormone (GH), Interleukin 6 (IL-6), Irisin, Tumor necrosis factor alpha (TNF-α), and receptor activator of NF-kB ligand (RANKL) serve as a fundamental mechanism for understanding muscle and bone metabolism [6,7]. It has been accepted that Wnt signaling regulates bone metabolism with its activation leading to bone formation, while its inhibition via Sclerostin and Dickopff-1 (DKK-1) downregulates bone formation. On the other hand, the activation of RANKL and pro-inflammatory cytokines (IL-6, TNF-α) further enhances bone loss through resorption. RANKL is also expressed in skeletal muscle and inhibits myogenic differentiation, leading to muscle loss [8]. The relationship between Sclerostin, inflammatory cytokines, and exercise has not been explored extensively [9].

Furthermore, muscle metabolism is primarily regulated by Irisin, a pro-myogenic factor derived from the fibronectin type III domain-containing protein 5 (FNDC5), attributed to skeletal muscle hypertrophy and increased protein synthesis via the AKT/mTOR pathway [8]. Several studies have reported an association between low circulating Irisin levels and low muscle and bone mass; however, its relationship with fat and lean body mass remains uncertain [10,11]. These various signaling pathways ultimately exert autocrine, paracrine, and endocrine effects on bone and muscle.

Although the concept of “inflammaging” was introduced a while ago, there is still a lack of research addressing this complex molecular network of bone, muscle, and inflammatory markers [12]. Evidence suggests that aging is associated with concomitant muscle and bone mass loss, resulting in sarcopenia and osteoporosis. In fact, evidence from animal and human studies support the reciprocity in understanding these conditions and shares common patterns of cellular dysregulation, including decreased estrogen hormone, increased inflammatory cytokine signaling (IL-6 and TNF-α), suppression of Wnt gene, and increased RANKL level. Age-related muscle loss, sarcopenia, is characterized by muscle mass and strength loss. Various factors influence sarcopenia, such as nutrition, physical activity/mechanical loading, and genetic components [13].

Moreover, it is essential to consider sex-based differences in hormone levels, muscle, and bone in exploring inflammaging. Certain age-related changes are mediated in a sex-specific manner that contribute to health risks, as evident where men appear to be better protected against age-related bone and muscle loss compared to women [12,14,15]. Consequently, comprehending sex-specific aging patterns will be instrumental in improving adverse metabolic and functional outcomes.

The benefits of physical activity throughout the lifespan have been well-accepted within the scientific community. However, only a few studies have explored the interaction of bone, muscle, and inflammatory markers simultaneously. Previous work reported that competitive physical activity was associated with serum levels of Irisin and RANKL but not with bone markers and Vitamin D levels, suggesting that myokine levels are related to the degree of muscle strength and volume, in the physical activity group compared to the control group [14]. Another study reported the inverse association between the Wnt inhibitor Sclerostin, expressed in higher levels in sarcopenic women with low skeletal muscle mass, suggesting the important linkage between muscle and bone via physical force [16]. Similarly, the cross-sectional study reported that serum IL-6 was inversely associated with skeletal muscle and bone parameters in women between 20–89 years, suggesting the importance of understanding the concept of inflammaging. However, this study did not examine other bone, muscle, or inflammatory marker parameters [15].

Although studies support the idea that mechanical signal/physical activity plays a tremendous role in muscle and bone health, the precise mechanisms for bone–muscle crosstalk are still not apparent. These observations led us to hypothesize about the role of RANKL, Irisin, IL-6, Sclerostin, Vitamin D, TNF-α and DKK-1 on muscle performance. This study aims to evaluate and compare bone and inflammatory markers between young men and women, as well as examine their relationship with muscle performance, to enhance understanding of the bone–muscle crosstalk mechanism. Therefore, recognizing the significance of these bone, muscle, and inflammatory markers aids in the development of novel screening methods for predicting adverse health events in clinical and sports-performance populations. Additionally, these markers can potentially be utilized to establish links with training regimens and monitor healthcare outcomes.

## 2. Methods

This study utilized a non-randomized, cross-sectional design with sex as an independent variable. We performed G*Power 3.1 analysis to estimate the sample sizes needed for 80% Power based on α = 0.05 and effect sizes (Cohen’s d_s_) for sex differences in Sclerostin and IL-6 [15,17]. We found a large effect size for gender (1.13, 1.42), requiring a sample size of 20 per group. A total of 40 college-aged students aged 18–25 years were screened for their eligibility. A total of 2 participants were lost in follow-up and were excluded; therefore, 38 healthy young men (*n* = 18) and women (*n* = 20) of diverse ethnicities (Caucasian, African American, and Asian) completed the protocols. All participants who were healthy and recreationally active, without cardiovascular and metabolic diseases or physical disabilities, and not taking any medications that affect muscle metabolism were included in this study. Participants were excluded if they had existing cardiovascular disease, uncontrolled hypertension, orthopedic pain, and physical disabilities in lifting weights. Only nine female participants reported the use of contraceptives. Participants were screened for inclusion/exclusion criteria before their first visit. All the participants were provided with the study protocol details outlining the risks and benefits of the study. Participants provided the written informed consent form before participating. All the protocols were approved by the Lander University Institutional Review Board.

### 2.1. Protocol

Participants completed two visits to the Human Performance Laboratory. During the first visit, participants completed written consent, a health history questionnaire, a menstrual history questionnaire, an international physical activity questionnaire (IPAQ), a bone-specific physical activity questionnaire (BPAQ) [18], and familiarization with the testing protocol. The validated long-form IPAQ was used to measure physical activity scores based on the metabolic equivalents (METs) of self-reported physical activities for seven days [19,20]. Participants also completed the Calcium intake questionnaire consisting of daily Calcium intake information based on certain foods consumed daily or weekly [21]. Further, lower- and upper-body strength were assessed using a vertical jump test and handgrip dynamometry, respectively. During the second visit, a blood sample was drawn to quantify the levels of Sclerostin, DKK-1, Irisin, IL-6, RANKL, TNF-α, and Vitamin D.

### 2.2. Muscular Performance

Prior to the neuromuscular performance test, each participant’s height and weight were measured using a wall-mounted stadiometer (Novel Products, Rockton, IL, USA) and a digital scale (Tanita Inc., Arlington Heights, IL, USA), respectively. The participant’s lower and upper body strength was assessed by the vertical jump test (Just Jump, Probotic, AL, USA) with a Tendo FitroDyne (Tendo Sports Machines, Trencin, Slovak Republic) and a handgrip (HG) test using handgrip dynamometry (Takei Scientific Instruments, Yashiroda, Japan), respectively. The participants were asked to step on the mat, stand with feet shoulder-width apart, and perform three countermovement vertical jumps, each separated by a minute rest. The three trials were averaged for the analyses. Average muscle power was estimated from the average force and Velocity reported by the Tendo machine. Relative Power was determined by dividing the average power by the body weight for each participant. Participants performed handgrip strength by flexing the elbow 0–30 degrees dorsiflexion and 0–15 degrees ulnar deviation in both dominant and non-dominant hands (3 trials on each side, each separated by 1 min rest). The grip width was individually adjusted to ensure comfort for each subject. Participants were instructed to squeeze as hard as possible for 3–5 s. The highest maximal handgrip strength for the right and the left hands were used for data analyses.

During the second visit, a fasting (>10 h) venipuncture blood sample (7 mL) was collected in the morning by a registered nurse at Lander University. The second visit was scheduled two days after the completion of the first visit. Participants were instructed to refrain from strenuous physical activity before the blood draw. All the serum samples were stored at −84 °C prior to the analysis of serum levels of Sclerostin, Dickkopf-1, Irisin, IL-6, Vitamin D, RANKL, and TNF-α. Commercial Enzymed Linked Quantikine Human SOST Immunoassay, Human IL-6 Immunoassay, and Human TNF-α Immunoassay (R and D system Inc., Minneapolis, MN, USA) were used to measure serum levels of Sclerostin, IL-6, in duplicate. A commercial Enzyme-Linked Human Biomedica Immunoassay Kit (Biomedica Medizinprodukte GmbH, Vienna, Austria) was used to measure serum levels of RANKL, Irisin, and DKK-1 in duplicate. Further, a commercial Enzyme-Linked Bioscience Human immunoassay kit was used to measure the serum level of Vitamin D in duplicate (Eagle Biosciences, Inc., Nashua, NH, USA). In this study, the intra-assay CVs for all the assays were less than 20%.

### 2.3. Statistical Analysis

**Figure 1:**
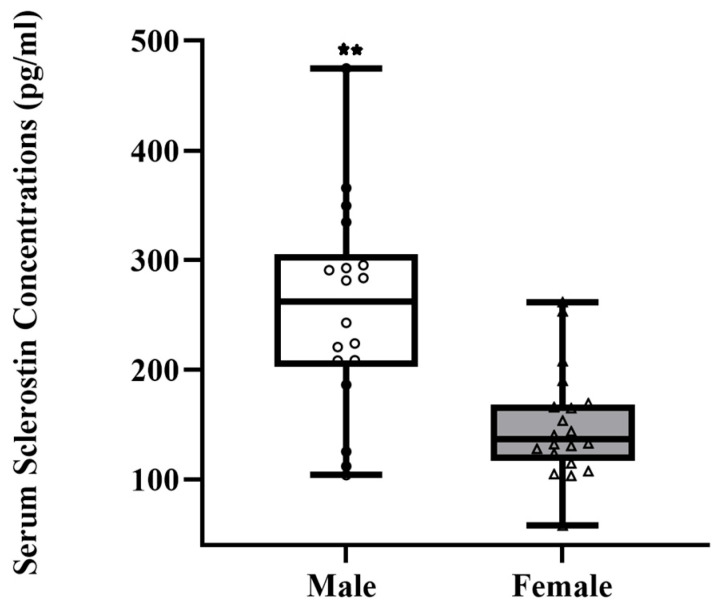
Boxplot showing a pattern of serum Sclerostin concentrations in males (*n* = 18) and females (*n* = 20). ** *p* < 0.001 significant sex difference. Effect size (Hedges’ g) = 1.40 (95% C.I. [0.69–2.09]).

**Figure 2:**
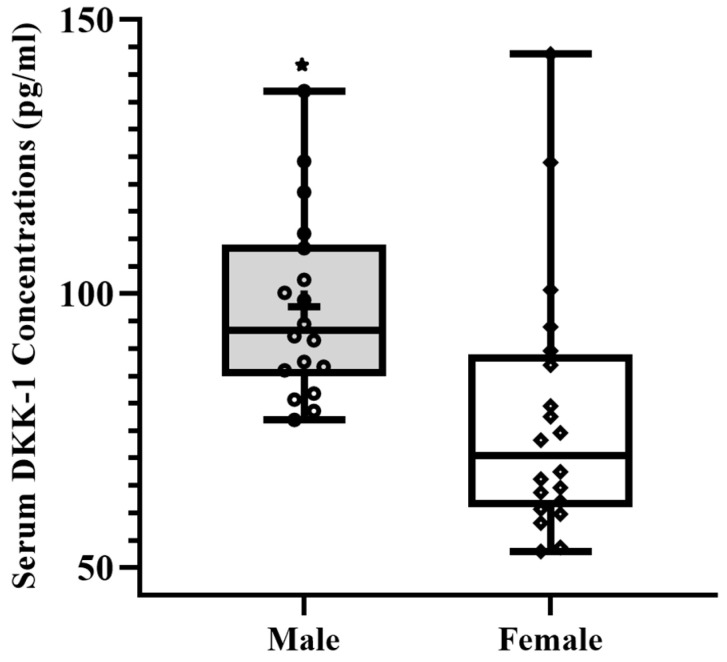
Boxplot showing a pattern of serum DKK-1 concentrations in males (*n* = 18) and females (*n* = 20). * *p* < 0.05 significant sex difference. Effect size (Hedges’ g) = 0.95 (95% C.I. [0.28, 1.6)).

**Figure 3:**
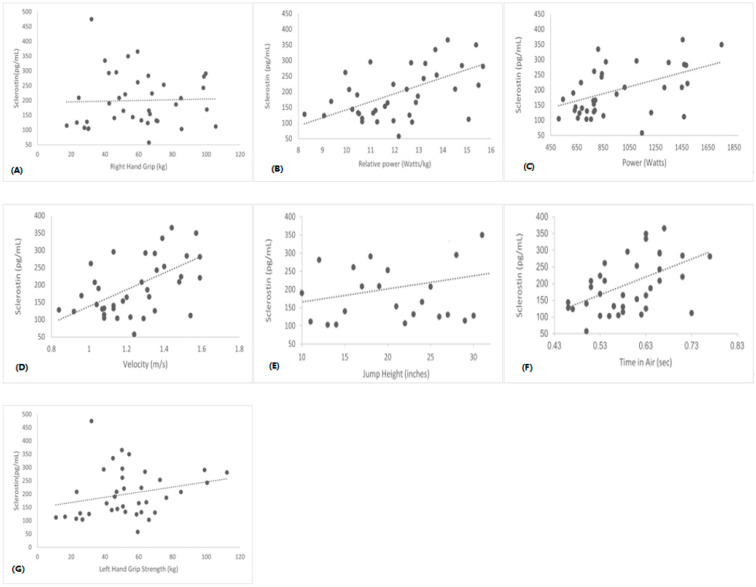
Correlations between serum Sclerostin vs. Right Hand Grip (Panel **A**) (r = 0.83; *p* = 0.62), Relative Power (Panel **B**) (r = 0.46 *p* = 0.03), Power (Panel **C**) (r = 0.48; *p* = 0.02), Velocity (Panel **D**) (r = 0.48; *p* = 0.02), Jump Height (Panel **E**) (r = 0.47; *p* = 0.03), Time in Air (Panel **F**) (r = 0.47; *p* = 0.03), and Left Hand Grip (Panel **G**) (r = 0.27; *p* = 0.09).

**Figure 4:**
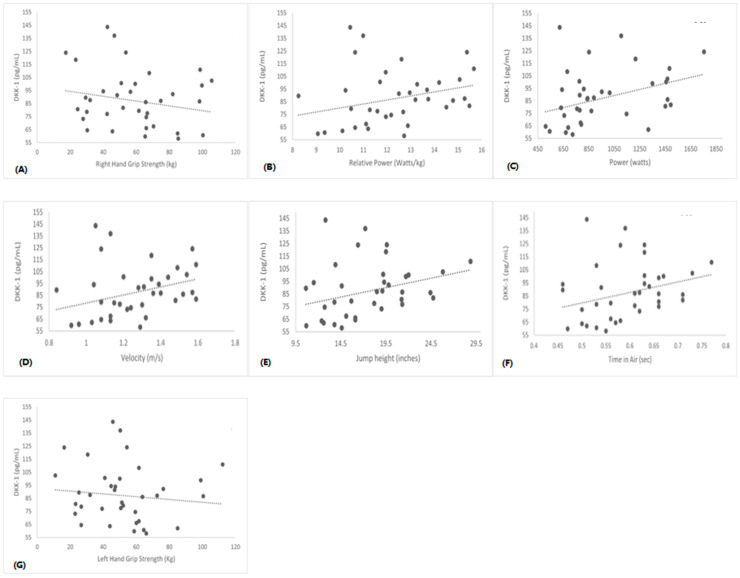
Correlations between serum DKK-1 vs. Right Hand Grip (Panel **A**) (r = −0.19; *p* = 0.23), Relative Power (Panel **B**) (r = 0.36; *p* = 0.02), Power (Panel **C**) (r = 0.39; *p* = 0.01), Velocity (Panel **D**) (r = 0.42; *p* = 0.00), Jump Height (Panel **E**) (r = 0.39; *p* = 0.01), Time in Air (Panel **F**) (r = 0.36; *p* = 0.02), and Left Hand Grip (Panel **G**) (r = −0.19; *p* = 0.24).

Data were analyzed using IBM SPSS 27.0 (SPSS Inc., Chicago, IL, USA). Unless otherwise stated, all descriptive statistics were reported as mean ± standard deviation (SD). All the dependent variables were tested for normality using the Kolmogorov–Smirnov test. Sex differences comparisons for non-normally distributed dependent variables (Sclerostin and DKK-1) were performed using the Mann–Whitney U test (Figure 1 and Figure 2). Independent t-tests were used to compare the physical characteristics of the two groups (Table 1). Independent t-tests were used to compare serum levels of Irisin, IL-6, RANKL, TNF-α and Vitamin D, and muscle performance variables. One-way ANCOVA adjusting for height and weight was used to determine those markers and muscle performance. Bonferroni-adjusted *p* values were used for the independent t-tests to avoid inflated type I error (Table 2 and Table 3). Pearson Product Moment Correlation coefficients (r) were computed to determine the relationships between bone and inflammatory markers and muscle performance variables for all 38 participants (Table 4). Correlations between non-normally distributed variables were computed using Spearman’s Rho (r_s_) (Figure 3 and Figure 4). We also included independent variables such as sex, age, BMI, IPAQ, BPAQ, CI, time in air, jump height, Velocity, Power, Relative Power, and grip strength variables in the stepwise regression models in determining if any of those independent variables predict bone, muscle, and inflammatory markers (Table 5). The levels of significance were set at *p* ≤ 0.05.

## 3. Results

Table 1 shows physical characteristics, body mass index, total bone physical activity questionnaire score, dietary Calcium intake, and total physical activity scores. Male and female participants had a significant difference in height, weight, and tBPAQ score. Figure 1 and Figure 2 show serum Sclerostin (male median = 261.91 pg/mL vs. female median = 136.51 pg/mL) and DKK-1 levels (male median = 93.32 pg/mL vs. female median 70.38 pg/mL) were significantly higher in male compared to female participants (*p* < 0.05). Data on bone, muscle, and inflammatory markers are presented in Table 2. Male participants shows higher serum Irisin (*p* < 0.05 Hedges’ g = 0.66, 95% C.I. [0.015, 1.30)) and Vitamin D (*p* < 0.05 Hedges’ g = 0.65 95% C.I. [0.005, 1.29)) than female participants. We found no significant difference in serum levels of IL-6, RANKL, and TNF-α (*p* > 0.05). Participants’ upper and lower body muscle performance was reported in (Table 3). A significant difference was found in lower-body muscle performance between male and female participants, which was significantly higher in male participants (*p* < 0.01). We found no significant difference in right and left-hand grip strength between the two groups. For the entire cohort (Table 4), we found a significant positive correlation between BMI and IL-6 (r = 0.4; *p* = 0.01). Vitamin D is positively associated with Time in the Air (r = 0.3; *p* = 0.02), Jump Height (r = 0.3; *p* = 0.03), and Velocity (r = 0.04; *p* = 0.08), whereas it is negatively associated with Relative Power (r = −0.04; *p* = 0.04). TNF-α was negatively associated with Time in the Air (r = −0.3; *p* = 0.04), Jump Height (r = −0.3; *p* = 0.04), and Power (r = −0.3; *p* = 0.05). We also found a significant positive association between RANKL and upper body strength (r = 0.5; *p* = 0.01). We did not find any association between Irisin and muscle performance.

**Figure 5:**
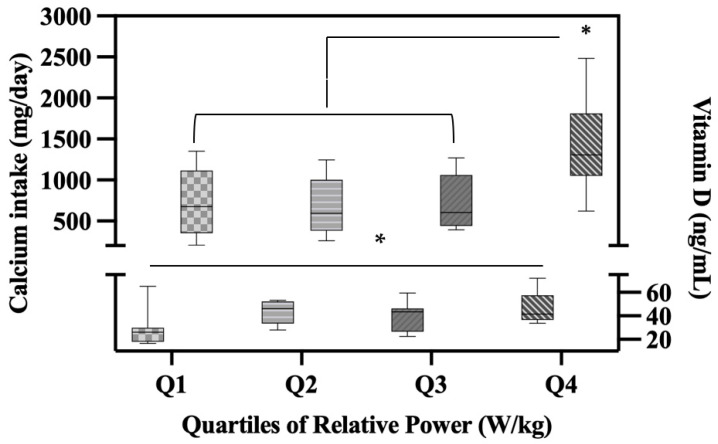
Boxplot showing Calcium Intake and Vitamin D levels across quartiles of Relative Power. * *p* < 0.05 significant. Effect sizes: Calcium intake μ^2^ = 0 (95% C.I. [0.08, 0.53]); Vitamin D μ^2^ = 0.25 (95% C.I. [0.005, 0.417)).

We also found a significant association between dietary Calcium intake and lower-body muscle performance variables, including Velocity (r = 0.42; *p* = 0.01), Power (r = 0.42; *p* = 0.009), and Relative Power (r = 0.47; *p* = 0.003). Due to this association between muscle performance variables, dietary Calcium intake, and Vitamin D, yet no significant associations between Vitamin D and Calcium intake, we conducted a gradient analysis on Calcium intake and Vitamin D across quartiles of Relative Power. We chose quartiles (Q) of Relative Power as the predictor variable due to its strong association with both Calcium intake and Vitamin D. One-way ANOVA revealed significant differences in Calcium intake (*p* = 0.001) and Vitamin D (*p* = 0.02) levels between quartiles of Relative Power. Calcium intakes were significantly higher in Q4 (1410.37 mg/day; SD = 566.82;) compared to the other quartiles (Q1 = 711.17 mg/day, SD = 409.53 *p* = 0.06; Q2 = 674.01 mg/day, SD = 333.70; *p* = 0.03; Q3 = 732.27 mg/day, SD = 330.48; *p* = 0.08). Additionally, Vitamin D levels were significantly higher in Q4 (46.71 ng/mL; SD 13.71;) compared to Q1 (27.80 ng/mL, SD 14.99; *p* = 0.01) (Figure 5).

Further, Spearman rho correlation analysis shows that Sclerostin and DKK-1 were positively associated with lower-body muscle performance (Figure 3 and Figure 4). We did not find significant correlations between serum Sclerostin and DKK-1 with upper-body muscle performance.

Table 5 shows stepwise regression analysis performed using sex, BMI, Calcium intake, tBPAQ, cBPAQ, total PA levels, Jump Height, Velocity, Power, Relative Power, and right and left handgrip to predict bone and inflammatory markers. We found that BMI is a 16% predictor of serum IL-6. Sex and Calcium intake account for 34–43% of serum sclerostin. Sex and tBAPQ account for 18% and 37% of serum DKK-, respectively. cBPAQ accounted for 14% of serum levels of Irisin. Relative Power, right-hand grip, and Calcium intake predict 20%, 32%, and 40% of serum levels of Vitamin D, respectively. Further, the right-hand grip predicted serum RANKL by 27%. Time in air accounted for 10% serum of TNF-α.

## 4. Discussion

The main findings from this study indicate that serum Sclerostin, DKK-1, Irisin, and Vitamin D levels are significantly different between male and female participants. Also, a significant difference was found only in lower-body muscle performance between the two groups. A positive relationship was found between Sclerostin and DKK-1 with lower-body muscle performance. Further, low to moderate relationships were found between RANKL, TNF-α, and Vitamin D with muscle performance variables.

Our findings of higher Sclerostin levels in male participants aligned with the previous studies [17,22], where serum Sclerostin levels were higher in male participants. We expected that Sclerostin levels would be higher in males, although the precise underlying mechanism remained unclear. One plausible explanation could be that a larger skeletal mass in men could primarily increase Sclerostin secretion in the bloodstream by osteocytes, assuming that circulating Sclerostin levels indicate the overall skeletal mass. However, it is important to note that this study did not assess the participant’s bone mineral density to account for the skeletal mass variations. It has also been reported that individuals who are highly active tend to have lower blood levels of Sclerostin compared to sedentary individuals [17]. We did not find any association between Sclerostin levels and physical activity scores, as all our participants were physically active based on their PA scores. In contrast to a previous study, our study reported a significant variation in serum DKK-1 levels, with higher levels observed in male participants [23]. This gender difference in DKK-1 levels potentially holds significance in relation to skeletal mass, health status, genetic factors, and hormonal variations, all of which can impact the outcome. Further investigation is warranted to delve into these findings. It is worth noting that while the previous study encompassed [23] a comparison between older and younger populations, our study solely focused on a cohort of healthy young individuals.

In Spearman’s correlation analysis, we found a significant moderate relationship between Sclerostin, DKK-1, and lower body muscle performance, which contrasts with the previous study [16]. The significance of circulating Sclerostin and DKK-1 in relation to skeletal mass remains unclear. The association between Sclerostin and muscle mass might be influenced by the interplay between bone mineral density and muscle mass rather than a direct link. Given the crosstalk of muscle, bone, and body fat, the correlation between Sclerostin, DKK-1, and muscle mass could be affected by body composition. Also, our study comprised a healthy population compared to previous findings from the sarcopenic population study [16]. We did not find significant differences between Hand Grip Strength, Sclerostin, or DKK-1.

In contrast, a previous study reported that Sclerostin levels are negatively associated with grip strength, possibly due to mechanical forces caused by gravity [24]. The inverse relationship could suggest that changes in Sclerostin levels could influence muscle activity, which could possibly increase serum Sclerostin levels during muscle atrophy or disuse. Also based on animal models, Wnt7a signaling stimulated the growth of skeletal muscles and enhanced muscle strength through the non-canonical pathway involving the activation of JNK or AKT-mTOR. These findings suggest the possibility of a similar implication in human studies in relation to muscle performance [25].

A growing body of evidence indicates that sarcopenia may impact metabolic abnormalities involving detrimental myokines and hormonal substances, such as Vitamin D, Irisin, IL-6, RANKL, and TNF-α [26]. In contrast to the previous study, we found that female participants had lower Irisin levels than males [27]. While a definitive explanation for this inconsistency is lacking, variations in population characteristics, such as age, body composition, and health status, could account for it. One hypothesis proposes that under normal metabolic conditions, skeletal muscle is the primary source of circulating Irisin, while in obese individuals, adipose tissue may produce and thus exert some influence on Irisin levels. In this study, we did not find significant differences in BMI between male and female participants [28]. Although BPAQ scores were widely reported as a predictor for bone mineral density [29], surprisingly, in this study, we found that the current BPAQ score is the predictor of serum Irisin levels accounting for 14% of the variance. Also, tBPAQ was negatively associated with right-hand grip strength (0.34), which might be related to upper-body muscle performance. There was also no correlation between Irisin and muscle performance variables, contrasting the previous studies [16,30]. These intriguing findings warrant further investigation. Several studies reported that exercise induces changes in Irisin levels [27,30]. However, based on IPAQ scores, we found no correlation between physical activity and circulating Irisin levels. While finding a long-term association between physical activity and Irisin levels is not anticipated, it is important to acknowledge the questionnaire that may have influenced these findings although the participants were physically active. It is important to note that while Irisin’s involvement in the browning of white fat cells and its impact on energy expenditure have been well studied, additional research is needed to identify its specific connections and interactions with bone and muscle.

We found significant differences in the serum Vitamin D levels between men and women in this study, although both groups showed sufficient Vitamin D levels, which agrees with the previous study [31]. Many factors affect Vitamin D status; however, in this study, we collected blood samples in the late Fall season. We also reported participant’s dietary Calcium intake analysis and found that female participants have lower Calcium intake compared to male counterparts, although the levels were not significant. Only a few studies have reported the relationship between Vitamin D levels and muscle performance. Interestingly, we found a significant correlation between Vitamin D serum levels and lower body muscle performance, in contrast to the previous studies [31,32]. Newer findings indicate that low Vitamin D levels are associated with decreased muscle mass in older adults; however, in our study, all the participants were young and physically active based on the IPAQ score. We also found Calcium intake, right-hand grip strength, and Relative Power as a predictor of Vitamin D levels.

Previous studies reported that elevated levels of circulating inflammatory mediators, such as TNF-α, IL-6, and RANKL, can contribute to muscle catabolism, ultimately reducing the mass and strength [33]. TNF-α has been shown to stimulate the production of additional catabolic cytokines, including IL-6, thereby triggering a subsequent cycle of inflammation. Contrary to the findings of previous studies [15,34,35], we did not observe significant gender differences in IL-6 and TNF-α levels. However, it should be noted that the participants in those studies were older compared to our study population. Interestingly, we found a significant association between IL-6 and BMI, aligning with one study [36], suggesting that the proinflammatory cytokines regulate adipose and skeletal muscle, which could be a way to differentiate between inflammation related to underlying disease and muscle recovery. A meta-analysis study reported that higher IL-6 is negatively associated with grip strength, which this study did not corroborate [37]. However, the population sample was among an older population compared to our study. Furthermore, we observed a negative relationship between TNF-α and lower-body muscle performance, which aligns with the previous study [36]. This suggests that cytokine levels could potentially influence muscle strength, which can have various effects on the body, even at a young age.

Additionally, we did not find a significant sex difference in serum levels of RANKL. Animal models suggest that overexpression of RANKL causes decreased muscle function, emphasizing its role in bone and muscle metabolism [8]. Additionally, anti-RANKL treatment has been reported to improve muscle inflammation and loss [38]. While limited research has explored the relationship between RANKL, muscle performance, and exercise performance in humans, our study identified a positive association between RANKL and hand grip strength, contrasting the findings from another study [39,40], which showed no significant association between hand grip strength and RANKL levels in healthy individuals, patients with heart failure and patients with inflammatory bowel disease [41]. Also, acute training exercise showed no changes in the serum RANKL level in college women, which aligns with the current study as all the participants are active based on the IPAQ score. The precise mechanism underlying this relationship remains unexplored; however, it is possible to hypothesize that RANKL initiates a signaling cascade and induces osteoclast differentiation in bone and muscle, inhibiting myogenic differentiation via NF-κB activation resulting in muscle loss [42]. Further, animal studies have suggested RANKL also contributes to decreased glucose uptake in the skeletal muscle and lowers the contractile properties of muscle function [43].

This study has certain limitations that should be taken into account. Our findings need to be interpreted within the context of the research design, which was cross-sectional in nature, as well as the comparisons made regarding physical activity and the relatively smaller sample size. This study reported correlations between biomarkers and muscle performance variables and did not establish causality. In this study, we did not control for the menstrual cycle during blood sampling since Sclerostin and DKK-1 levels were not affected during the menstrual cycle [44]; however, it should be noted that inflammatory markers could be affected by menstrual cycle [45]. We did not measure bone mineral density or muscle cross-sectional area, which could have provided additional insights and expanded the scope of the results. Furthermore, the assessment of physical activity levels relied on self-reported questionnaires, which may introduce some subjectivity. It is worth considering the possibility of exploring exercise interventions in relation to the current variables in the future studies to enhance our understanding of this area further.

## 5. Conclusions

In conclusion, our results support the notion that there is a sex disparity for Sclerostin, DKK-1, Irisin, and Vitamin D levels. Further, we also found an association between muscle performance and bone, muscle, and inflammation markers. Extensive research exists regarding the interaction between bone and muscle; however, several crucial aspects remain to be addressed. Most of the studies have predominantly focused on one-way communication between bone and muscle. In contrast, in our study, we delineate the difference between sex and muscle performance, aiming to identify common bone and inflammatory markers; comprehending bone and muscle crosstalk as a complex endeavor and numerous unexplored areas necessitates further investigation. Additionally, this study provides insights into the concepts of “Exerkines” and “Inflammaging”, and their counterregulatory roles in inflammation, bone, and muscle metabolism. While inflammaging is typically associated with aging, our study, focusing on a young population, provides a unique perspective. Exploring these potential biomarkers allows us to assess individual fitness levels and disease prognosis more effectively at a young age.

## Figures and Tables

**Table 1:** Physical characteristics of the participants (Mean ± SD).

Variables	Male (*n* = 18)	Female (*n* = 20)
Age (years)	20.6 ± 1.6	20.6 ± 1.3
Height (cm)	178.0 ± 8.6	160.7 ± 6.9 **
Weight (kg)	85.1 ± 18.7	70.4 ± 18.5 *
Body Mass Index (kg/m^2^)	26.7 ± 4.4	27.1 ± 5.79
tBPAQ	21.5 ± 13.1	38.2 ± 29.8 *
Calcium Intake (mg/dL)	1015.4 ± 520.7	744.5 ± 465.9
Total PA Score (MET-min/Wk)	4743. 9 ± 3410.9	5781.32 ± 4893.7

* *p* < 0.05 Significant; ** *p* < 0.01 Significant tBPAQ = total Bone Specific Physical Activity Questionnaire; PA = physical Activity.

**Table 2:** Bone, muscle, and inflammatory markers of the participants adjusted (Mean ± SD).

Variables	Male (*n* = 18)	Female (*n* = 20)
Irisin (ng/mL)	154.4 ± 7.2	130.3 ± 6.8 *
Vitamin D (ng/mL)	44.7 ± 3.3	34.7 ± 3.1 *
IL-6 (pg/mL)	3.69 ± 0.7	5.0 ± 0.6
RANKL (pg/mL)	4.2 ± 0.6	5.7 ± 0.6
TNF-α (pg/mL)	15.0 ± 1.4	15.0 ± 1.3

* Significant *p* < 0.05.

**Table 3:** Muscle performance variables (Mean ± SD).

Variables	Male (*n* = 18)	Female (*n* = 20)
Time in air (s)	0.6 ± 0.0	0.5 ± 0.0 **
Jump Height (inches)	20.3 ± 0.8	14.9 ± 0.7 **
Velocity (m/s)	1.4 ± 0.0	1.1 ± 0.0 **
Power (watts)	1075.6 ± 31.6	848.6 ± 29.9 **
Relative Power	13.6 ± 0.3	10.9 ± 0.3 **
Right-Hand Grip Strength (kg)	58.1 ± 6.0	59.7 ± 5.6
Left-Hand Grip Strength (kg)	52.8 ± 5.7	52.0 ± 5.4

** Significant *p* < 0.01

**Table 4:** Correlation between bone and inflammatory markers and muscle performance variables (*n* = 38).

Variables	IL-6	Irisin	RANKL	TNF-α	Vitamin D
BMI	r = 0.4 *	r = 0.3	r = −0.0	r = −0.2	r = 0.1
	*p* = 0.01	*p* = 0.8	*p* = 0.6	*p* = 0.1	*p* = 0.3
CI (mg/day)	r = −0.2	r = 0.0	r = 0.2	r = −0.0	r = −0.2
	*p* = 0.8	*p* = 0.5	*p* = 0.2	*p* = 0.7	*p* = 0.8
TIA (s)	r = −0.1	r = −0.2	r = 0.0	r = −0.3 *	r = 0.3 *
	*p* = 0.3	*p* = 0.2	*p* = 0.7	*p* = 0.04	*p* = 0.02
JH (inches)	r = −0.1	r = −0.2	r = 0.0	r = −0.3 *	r = 0.3 *
	*p* = 0.4	*p* = 0.1	*p* = 0.7	*p* = 0.04	*p* = 0.03
Vel (m/s)	r = −0.2	r = −0.2	r = −0.0	r = −0.1	r = 0.4 *
	*p* = 0.1	*p* = 0.2	*p* = 0.6	*p* = 0.4	*p* = 0.08
Power (watts)	r = −0.0	r = −0.7	r = −0.0	r = −0.3 *	r = 0.24
	*p* = 0.7	*p* = 0.6	*p* = 0.7	*p* = 0.05	*p* = 0.1
R. Power	r = −0.2	r = −0.1	r = −0.0	r = −0.2	r = −0.4 *
	*p* = 0.1	*p* = 0.2	*p* = 0.7	*p* = 0.1	*p* = 0.04
RH Grip S (kg)	r = −0.2	r = 0.0	r = 0.5 *	r = 0.1	r = −0.2
	*p* = 0.2	*p* = 0.8	*p* = 0.01	*p* = 0.4	*p* = 0.1
LH Grip S (kg)	r = −0.1	r = 0.8	r = 0.4 *	r = 0.0	r = −0.2
	*p* = 0.3	*p* = 0.6	*p* = 0.01	*p* = 0.6	*p* = 0.1

* Significant *p* < 0.05; BMI—Body Mass Index; CI—Calcium Intake; TIA—Time in air; JH—Jump height; Vel—Velocity; R. Power—Relative Power, RH Grip S—Right hand grip strength; LH Grip S—Left hand grip strength.

**Table 5:** Regression analysis results for serum IL-6, serum Sclerostin, serum DKK-1, serum Irisin, serum Vitamin D, serum RANKL, and serum TNF-α.

Dependent Variable	Predictor Variables	β	SEE	R^2^	*p*
IL-6 *	BMI	0.23	2.78	0.16	<0.014
Sclerostin *	Gender Calcium Intake	−92.40 0.05	75.60 71.64	0.34 0.43	<0.032
DKK-1 **	Gender tBPAQ	−26.37 0.47	20.85 18.04	0.18 0.37	<0.001
Irisin *	cBPAQ	−0.42	29.38	0.14	<0.018
Vitamin D *	Relative Power Right Hand Grip Calcium Intake	4.71 −0.20 −0.009	12.82 12.01 11.48	0.20 0.32 0.40	<0.020
RANKL **	Right Hand Grip	0.60	2.38	0.27	<0.001
TNF-α *	Time in Air	−25.10	5.63	0.10	<0.046

β-Standardized Regression Coefficient; * *p* < 0.05; ** *p* < 0.01.

## Data Availability

Not applicable.
